# Deletion of the PA4427-PA4431 Operon of *Pseudomonas aeruginosa* PAO1 Increased Antibiotics Resistance and Reduced Virulence and Pathogenicity by Affecting Quorum Sensing and Iron Uptake

**DOI:** 10.3390/microorganisms9051065

**Published:** 2021-05-14

**Authors:** Lixin Shen, Lang Gao, Mengjiao Yang, Jian Zhang, Yulu Wang, Yuqi Feng, Liping Wang, Shiwei Wang

**Affiliations:** Key Laboratory of Resources Biology and Biotechnology in Western China, School of Life Sciences, Northwest University, Ministry of Education, Xi’an 710069, China; shenlx@nwu.edu.cn (L.S.); gaolang0814@163.com (L.G.); 201920931@stumail.nwu.edu.cn (M.Y.); 201731840@stumail.nwu.edu.cn (J.Z.); wangyulu@stumail.nwu.edu.cn (Y.W.); 201831864@stumail.nwu.edu.cn (Y.F.); wanglipingmay@163.com (L.W.)

**Keywords:** respiratory chain, cytochrome *bc1* (cyt*bc1*), antibiotic resistance, virulence and pathogenicity, quorum-sensing systems, iron transport

## Abstract

The respiratory chain is very important for bacterial survival and pathogenicity, yet the roles of the respiratory chain in *P. aeruginosa* remain to be fully elucidated. Here, we not only proved experimentally that the operon PA4427-PA4431 of *Pseudomonas aeruginosa* PAO1 encodes respiratory chain complex III (cyto*bc1*), but also found that it played important roles in virulence and pathogenicity. PA4429–31 deletion reduced the production of the virulence factors, including pyocyanin, rhamnolipids, elastase, and extracellular polysaccharides, and it resulted in a remarkable decrease in pathogenicity, as demonstrated in the cabbage and *Drosophila melanogaster* infection models. Furthermore, RNA-seq analysis showed that PA4429–31 deletion affected the expression levels of the genes related to quorum-sensing systems and the transport of iron ions, and the iron content was also reduced in the mutant strain. Taken together, we comprehensively illustrated the function of the operon PA4427–31 and its application potential as a treatment target in *P. aeruginosa* infection.

## 1. Introduction

The respiratory chain, including the aerobic and anaerobic respiratory branches, is one of the most key supplying energy pathways for cellular metabolism. The shared main respiratory equipment of the aerobic and anaerobic respiration pathways contains NADH dehydrogenase (complexes I), coenzyme Q, cytochrome *bc*1 complex (complex III), and cytochrome *c*. In addition, in *Pseudomonas aeruginosa*, the aerobic chain contains terminal oxidases (complex IV, e.g., panthenol and cytochrome *c* oxidases), and the anaerobic respiratory chain possesses a series of NOx reductases (e.g., nitrate, nitrite, nitric oxide, and nitrous oxide reductases) [1]. Protons are pumped out across the bacterial membrane during electron transfer through complexes I, III, and IV, which form the proton gradient for ATP synthesis [2]. The *bc*1 complex in the center of the respiratory chain is responsible for the electrons transferring from coenzyme Q to cytochrome *c* [3]. All *bc*1 complexes contain three redox subunits: *cyt b* (two *b*-type hemes: *b*_L_ and *b*_H_), cyt *c*1 (a *c*-type heme), and ISP (two Fe-S clusters) [4].

Since the respiratory chain is indispensable in the energy metabolism of all living organisms, mutation of the respiratory chain complexes can lead to various severe physiological defects [5]. For example, damage to the mitochondrial respiratory chain complex [6] results in membrane potential loss [7], a reduction of ATP production [8], age-related neurodegenerative and metabolic syndrome, and even cancer [6]. The respiratory chain has also been reported to play a crucial role in the viability and virulence of bacteria. Functionality impairment of the respiratory chain causes a rapid loss of cell viability in *Mycobacterium tuberculosis* [9]. Inactivation of complex I of the respiratory chain disrupts the aerobic respiration and virulence of some microbes, like *Streptococcus agalactiae* [10], *S. sanguinis*, and *S. pneumoniae* [11,12].

The importance of the respiratory chain’s role makes it the main target of various natural and synthetic antimicrobial drugs for both eukaryotic and prokaryotic cells [13]. Several compounds have been reported to be complex III inhibitors in some eukaryotes, like atovaquone against *Toxoplasma gondii* and *Pneumocystis sp*. and 2-hydroxy-naphthoquinone, pyridones, quinolones, acridones, acridine diones, and rhinacanthin against malaria parasites [14]. Amilorides, the well-known inhibitors of Na^+^/H^+^ and Na^+^/Ca^2+^ antiporters and Na^+^ channels, have also been reported to be able to inhibit bacterial and mitochondrial NADH-quinone oxidoreductase (complex I) [15]. Cytochrome *bc*1 of *M. tuberculosis* is inhibited by a new small molecule substance Q203, which has become a clinical drug target for *M. tuberculosis* infection treatment [16].

*P. aeruginosa*, a ubiquitous Gram-negative bacterium, is able to survive in various environments, such as soils, waters, or medical device surfaces [17]. It rarely affects healthy individuals but causes high morbidity and mortality in cystic fibrosis (CF) patients and immunocompromised individuals [18]. Treatment of *P. aeruginosa* infections has become a great challenge, due to its high innate and acquired resistance to many available antibiotics [19]. Discovery of new specific drug targets is helpful for *P. aeruginosa* infection treatment. The multibranched respiratory chain could be a potential target of *P. aeruginosa* infection treatment because it not only produces more than 90% of cellular ATP but also enhances the adaptability of this bacterium [2,14]. According to genomic sequence analysis, there are at least 17 kinds of dehydrogenases [20], 3 kinds of cytochrome *c*, and 5 kinds of terminal oxidase in its genome [21]. Among them, the genes PA4429-PA4431 have been predicted to code cytochrome *bc*1, according to the KEGG pathway database (https://www.kegg.jp/kegg/pathway.html) (accessed on 1 May 2019) [22] and the *P. aeruginosa* genome database (http://www.pseudomonas.com) (accessed on 1 May 2019) [23]. However, very limited experimental evidence is available regarding the identification of cyt*bc*1 in *P. aeruginosa* PAO1, as well as its roles in pathogenicity.

In our previous work, a mutant with a threefold increased resistance to aminoglycoside antibiotics was screened out from a random transposon mutant library [24]. In this report, the transposon insertion site was found to be located in the gene PA4431. PA4429–31 were experimentally demonstrated to encode cyt*bc*1. Next, the effects of PA4427–31 on virulence and pathogenicity were investigated by cabbage and *Drosophila melanogaster* infection models, and the reasons for the reduction of pathogenicity were illustrated by RNA-Seq analysis and molecular experiments. Taken together, all the genetic and biochemical evidence proved the important contributions of cyt*bc*1 to the virulence and pathogenicity of *P. aeruginosa* PAO1, indicating that it might be used as a potential therapeutic target for *P. aeruginosa* infection.

## 2. Materials and Methods

### 2.1. Bacterial Strains and Growth Conditions

The *P. aeruginosa* and *Escherichia coli* strains listed in the Table 1 were routinely cultured using Luria–Bertani (LB) agar plates or broth at 37 °C. In addition, *Pseudomonas* isolate agar (PIA) plates were also used for *P. aeruginosa* growth. When necessary, tetracycline (Tc, 15 μg/mL), kanamycin (Kan, 50 μg/mL), or gentamicin (Gm, 15 μg/mL) were used for *E. coli*, and tetracycline (Tc, 300 μg/mL), gentamicin (Gm, 150 μg/mL) or carbenicillin (Cb, 250 μg/mL) was used for *P. aeruginosa*. All antibiotics used were purchased from Amresco (Solon, PA, USA).

### 2.2. RT-PCR

The various mutants were grown in LB broth, and the total RNA was extracted using an RNA extraction kit (RNA prep Pure Cell/Bacteria Kit, TIANGEN, Beijing, China). Then, 1 μg of RNA and random PCR primers (Table 2) were used to synthesize cDNA with a Prime Script TM RT reagent kit (TaKaRa, Dalian, China) according to the manufacturer’s instructions. To determine the operon profile, different primer sets were designed and used for PCR (Table 2), and the non-retrotranscribed mRNA was used as a negative control.

### 2.3. Construction of Mutants

A *sacB*-based method was used to construct the in-frame deletion mutants [25]. Taking PAO1(ΔPA4429) as an example, the 0.78 kb upstream fragment of PA4429 was amplified using a forward primer PA4429-UP-S and a reverse primer PA4429-UP-A containing *Eco*R I and *Sac* I restriction sites, respectively (Table 2). The 0.75 kb downstream region of PA4429 was generated with the forward primer PA4429-D-S and the reverse primer PA4429-D-A containing the *Sac* I and *Kpn* I sites, respectively. The upstream and downstream fragments of PA4429 were digested and then successively ligated into pEX18Tc to generate the plasmid pEX18Tc-PA4429. PAO1(ΔPA4429) was obtained by triparental mating as reported previously [26]. In brief, PAO1, *E. coli* DH10B containing pEX18Tc-PA4429, and *E. coli* DH10B containing pRK2013 were cultured overnight, collected, washed with PBS, and finally suspended in SOC medium. The bacteria were mixed at a ratio of 1:1:1 in a microcentrifuge tube and then spotted onto a LB agar plate. After 12 h of growth at 37 °C, the bacteria were resuspended in 500 mL of LB medium and then plated onto two PIA plates with a tetracycline addition. After being cultured on 10% sucrose-containing PIA agar, the resultant mutants were selected and verified by PCR.

### 2.4. Complementation of the In-Frame Deletion Mutants

To complement the PA4429 deletion, PA4429, PA4429–31, and PA4427–31 were respectively PCR amplified and ligated into the pAK1900 vector (Biovector NTCC Inc. Beijing, China). Taking PAO1(ΔPA4429)C1 as an example, PA4429 was PCR amplified by the forward primer and the reverse primer with the *Hin*d Ⅲ and *Bam* HⅠ restriction sites, respectively. The fragment was ligated into pAK1900 to form a new plasmid pAK4429, which was next transferred into *E. coli* DH10B. The transfer of pAK4429 into the PAO1(ΔPA4429) was performed by electroporation. The integrants were selected on PIA plates containing 250 μg/mL Cb. The resultant strain was named PAO1(ΔPA4429)C1. Similarly, PAO1(ΔPA4430)C1, PAO1(ΔPA4430)C2, PAO1(ΔPA4430)C3, PAO1(ΔPA4431)C1, PAO1(ΔPA4431)C2, PAO1(ΔPA4431)C3, PAO1(ΔPA4429–31)C1, PAO1(ΔPA4429–31)C2, and PAO1(ΔPA4429–31)C3 were respectively constructed (Table 1).

### 2.5. The Disk Diffusion and Antibiotics Susceptibility Tests

For disk diffusion test, 10 μL of the 50 μg/mL antibiotic was spotted on filter disks with a 6-mm diameter, which were placed on LB agar plates with 40 μL of bacterial cells with 0.5 OD_600_. The plates were incubated at 37 °C for 24 h. The diameters of the inhibition zones around the antimicrobial agents were observed [27].

The minimum inhibitory concentrations (MICs) of the antibiotics were determined by twofold dilutions of the bacterial culture in 96-well plates. In brief, overnight bacterial cultures were diluted at 1:150 in LB, added to the wells containing different concentrations of antibiotics and incubated at 37 °C for 24 h. The MICs were recorded as the lowest concentrations of the antibiotics by measuring the growth after 24 h of incubation at 37 °C.

### 2.6. Biofilm Assay

The biofilm formation of the various mutant strains was compared with the wild-type strain, as previously described in [28]. In brief, after the various mutant strains were grown in LB overnight, 1 μL of culture was inoculated into 99 μL of LB broth in a 96-well polyvinyl chloride plate (Corning-Costar, NY, USA) and kept at 37 °C for 12 h. After the 96-well plate was stained by using 0.1% crystal violet for 10 min and washed 5 times with water, it was visualized and compared.

### 2.7. ATP Measurement

The amount of ATP was measured by the luciferin luciferase method, following the protocol of the ATP detection kit (Beyotime, Beijing, China) [29]. After being cultured in LB medium for 10 h, PAO1, PAO1(Δ4429–31) and PAO1(Δ4429–31)C were adjusted to an OD_600_ value of 1.0. Then, 2 mL of each cell culture was collected and centrifuged at 12,000 rpm for 5 min. The pellets were treated with 200 μL of a lysis buffer from the ATP detection kit. After being centrifuged at 13,000 rpm for 5 min at 4 °C, the supernatant was transferred to a new centrifuge tube for ATP testing. The luminescence from a 100 μL sample was assayed in a Victor2 multilabel counter (Wallac model 1450, Perkin-Elmer, Waltham, MA, USA) together with 100 μL of an ATP detection buffer from the ATP detection kit [30].

### 2.8. Assays of Pyocyanin, Rhamnolipid, Extracellular Polysaccharide, Elastase, and Motility

Pyocyanin was extracted from the culture supernatants of PAO1, PAO1(Δ4429–31), and PAO1(Δ4429–31)C and measured as previously described in [31]. Briefly, 3 mL of chloroform was added to 5 mL of the culture supernatant, vigorously vortexed for 2 min, and then centrifuged for 10 min at 8,000 rpm. After extraction, the chloroform layer was transferred to a fresh tube and mixed with 1 mL 0.2 M HCI. Then, the top layer was removed, and the OD_520_ was measured. The amount of pyocyanin, in μg/mL, was calculated using the following formula: OD_520_/OD_600_ × 17.072 =μg of pyocyanin per mL.

The rhamnolipid was analyzed according to the previous report [32]. For rhamnolipid extraction, *P. aeruginosa* PAO1, PAO1(Δ4429–31), and PAO1(Δ4429–31)C were grown at 37 °C for 24 h in LB medium. After incubation, the bacterial culture was centrifuged at 10,000 rpm for 5 min. The cell-free supernatant was mixed with an equal volume of ethyl acetate and vigorously vortexed to obtain rhamnolipid in its organic phase. The rhamnolipid extract was dissolved in 4 mL chloroform and put in 200 µL of freshly prepared 1 g/L methylene blue solution (pH 8.6) and 4.9 mL of distilled water. After vortexing for 4 min and standing for 15 min, the absorbance of the solution at 638 nm was measured, and the values were converted to rhamnolipid concentrations using a calibration curve.

An extracellular polysaccharide assay was performed by examining the colony color in the Congo Red agar plates (1% tryptone, 1% agar, 4% Congo Red, and 2% Coomassie brilliant blue) [28]. After being grown overnight, 2 µL of culture was inoculated in the plates and kept at 37 °C overnight. The plates were visualized and analyzed.

Elastase examination was carried out according to the previous method [33]. The overnight culture in LB was centrifugated, and 1 mL of supernatant was added to a new tube containing 5 mg Elastin-Congo Red and 2 mL of a 0.1 M phosphate buffer (pH 7.0) (Sigma, Saint Louis, MO, USA). After being kept at 37 °C for 3 h, the absorbance at 495 nm was measured.

The motility was assayed in different media [28]. Briefly, for the swarming and swimming test, 2 µL of overnight culture in LB broth was inoculated in the swarming (0.8% BD nutrient broth, 0.5% glucose, and 0.5% agar) and swimming (1% tryptone, 0.5% yeast extract, 1% NaCl, and 0.3% agar) plates, respectively. After keeping the overnight culture at 37 °C, the swarming and swimming zones were examined. The twitching motility was examined by stab-inoculating strains through a thin LB (1% tryptone, 0.5% yeast extract, 1% NaCl, and 1% agar) plate. After being kept at 30 °C for 24–48 h, the twitching zones were visualized at the agar plate interface by using 0.1% crystal violet.

### 2.9. In Vivo Virulence Assays

The virulence of PAO1 and PAO1(Δ4429–31) was investigated with cabbage (*Brassica pekinensis*) and fruitfly (*Drosophila melanogaster* Canton S) infection models, as reported previously in [34]. In brief, for the cabbage infection model, bacterial strains were cultured overnight in M9 broth (22 mM Na_2_HPO_4_, 22 mM KH_2_PO_4_, 85 mM NaCl, 0.1% NH_4_Cl, 2 mM MgSO_4_, 0.1 mM CaCl_2_, and 0.4% glucose) at 37 °C. Cells were collected and rinsed with 10 mM MgSO_4_. After adjusting the OD_600_ to 0.5, 10 mL of the dilution was injected into the stems with a micropipette [26]. The cabbage leaves were incubated at room temperature and assessed daily for visual signs of infection. For the fruit fly feeding model [35], the overnight cultures’ OD_600_ values were adjusted to 2.0 using M9 medium. The pellets from the 1.5 mL cultures were suspended in 100 µL of 5% sucrose. The suspensions were spotted on the sterile filter on the bottom of the culture flask. After cultivation at 37 °C for 30 min, 20 male flies anesthetized with carbon dioxide were sorted and placed in each flask. A PBS buffer was used as a negative control. The number of live fruit flies was counted at 24 h intervals.

### 2.10. RNA-Seq and Differentially Expressed Genes (DEGs) Analysis

The overnight culture of PAO1 and PAO1(Δ4429–31) was transferred to fresh LB broth until reaching an OD_600_ of 0.6–0.8, respectively. After being washed once with sterilized fresh PBS, the bacterial cells were quickly frozen with liquid nitrogen for sequencing. The RNA-Seq was carried out on an Illumina HiSeq TM 2500 (Gene Denovo Biotechnology, Guangzhou, China). Three independent experiments were performed. The DEGs of PAO1 and PAO1(Δ4429–31) were identified using the edgeR package (http://www.r-project.org/) (accessed on 1 June 2020) in R. Genes with a fold change ≥2 and a false discovery rate (FDR) <0.05 were treated as significant DEGs.

### 2.11. The Expression Level Measurement of the Genes Related to Quorum Sensing and Iron Absorption

The vector pMS402 was used to detect gene expression, as reported previously in [26]. The promoter region of the target genes was amplified and fused with the *luxCDABE* gene in the vector. In the assay, the overnight culture of the reporter strains was diluted at a 1:300 ratio in LB media, and 90 µL of these solutions were added to the wells of a 96-well black plate. The luminescence and OD_595_ values were measured every 0.5 h for a total of 24 h using a Victor2 multilabel counter (Wallac model 1450, Perkin-Elmer, MA, USA).

### 2.12. Measurement of Intracellular Iron Concentration

PAO1 and PAO1(Δ4429–31) were inoculated and cultured in 100 mL of LB liquid medium overnight. After centrifugation, the cell sediment was washed with a PBS solution 2–3 times and digested with 65% nitric acid for 30 min. The treated solution was diluted to 3%, and the iron concentration was measured through ICP-MS (Agilent, Santa Clara, CA, USA).

### 2.13. Statistical Analysis

Statistical analysis was performed using the computer software program SPSS version 18 for descriptive statistics including frequencies and the cross-tabulation of microbiological data. *P* < 0.05 was considered to be statistically significant.

## 3. Results

### 3.1. Deletion of PA4429–31 Increased Aminoglycoside Antibiotics Resistance

**Figure 1:**
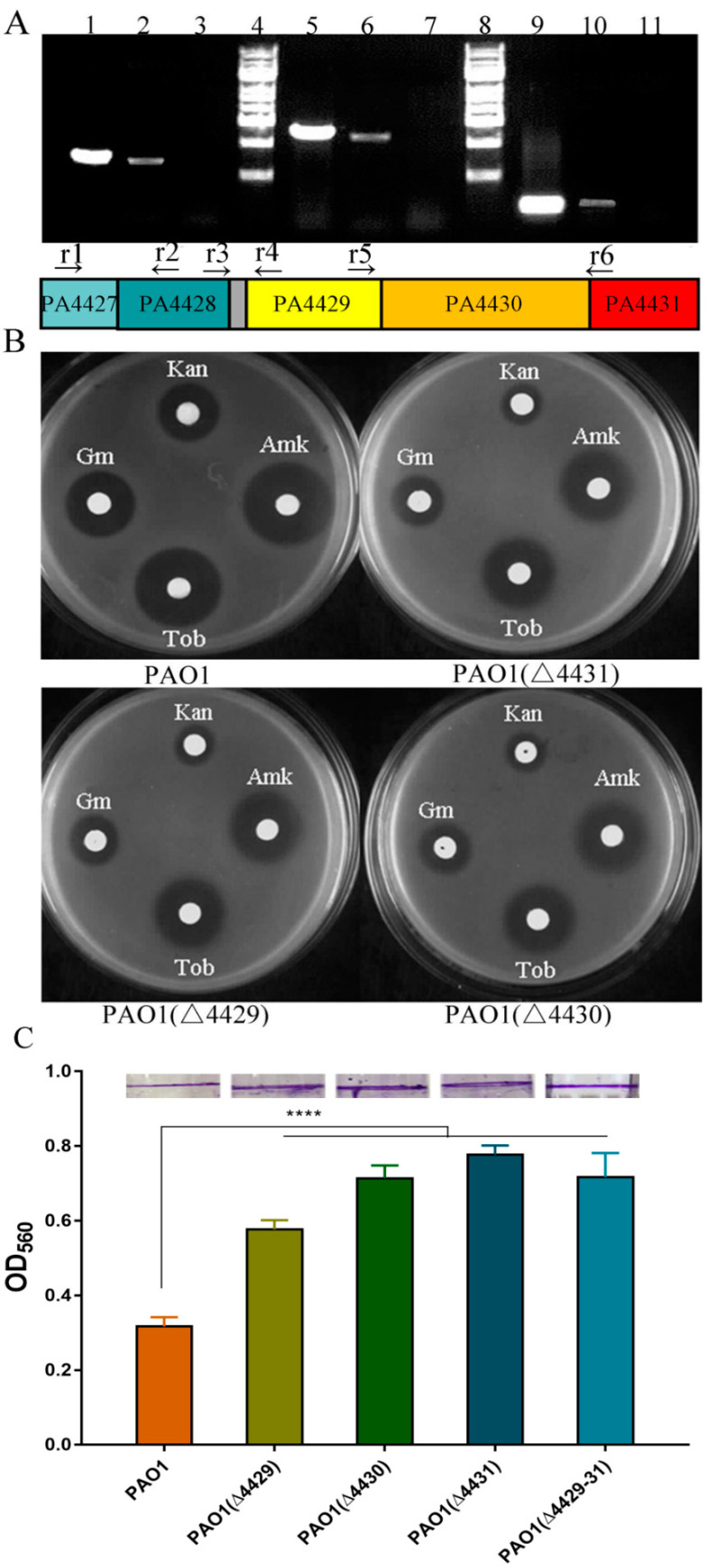
The transcription profiles of PA4427-PA4431 and the antibiotic susceptibility change after their mutation. (**A**) The products of PCR and RT-PCR using different primers and templates. The gray box between PA4428 and PA4429 is the intergenic region. Lane 1: r1 and r2 primers and genomic DNA template; Lane 2: r1 and r2 primers and cDNA template; Lane 5: r5 and r6 primers and genomic DNA template; Lane 6: r5 and r6 primers and cDNA template; Lane 9: r3 and r4 primers and genomic DNA template; Lane 10: r3 and r4 primers and cDNA template; Lanes 3, 7, and 11: non-retro-transcribed mRNA template as a negative control; and Lanes 4 and 8: 1 kb ladder marker. (**B**) Antibiotic susceptibility of PAO1, PAO1(Δ4429), PAO1(Δ4430), and PAO1(Δ4431) in LB plates with different antibiotics. Kan: kanamycin; Amk: amikacin; Gm: gentamicin; Tob: tobramycin. (**C**) The biofilm was examined in the PAO1, PAO1(ΔPA4428), PAO1(ΔPA4429), PAO1(ΔPA4430), PAO1(ΔPA4431), and PAO1(ΔPA4429–31) strains. The error bars represent standard errors. **** significantly different, *p* < 0.001.

A transposon mutant with a threefold increase of aminoglycoside antibiotics resistance was screened from our transposition mutant library [24]. The transposition insertion site was determined to be located in the PA4431 gene by arbitrary PCR and sequencing. The genes PA4427, PA4428, PA4429, PA4430, and PA4431 were predicted to constitute an operon according to the *P. aeruginosa* genome database (http://www.pseudomonas.com) (accessed on 1 May 2019), and it was consistent with the RT-PCR results (Figure 1A). To further investigate the contribution to the aminoglycoside antibiotics resistance of the individual genes, the in-frame deletion mutants of PA4429, PA4430, PA4431, and PA4429–31 were constructed and named as PAO1 (ΔPA4429), PAO1 (ΔPA4430), PAO1 (ΔPA4431), and PAO1 (ΔPA4429–31), respectively. The disk diffusion test showed that, compared with the wild-type strain PAO1, all the mutants exhibited smaller inhibition zones under the treatment condition of four kinds of the tested aminoglycoside antibiotics, including kanamycin, gentamycin, tobramycin, and amikacin (Figure 1B). Furthermore, their MIC values were determined, and the results showed that the MIC values of the single-gene deletion or the three-gene deletion were all increased threefold for the four antibiotics, indicating a resistance increase for the aminoglycoside antibiotics of these mutants (Table 3). In addition, the single-gene deletion and the three-gene deletion both exhibited an increase in the biofilm formation ability (Figure 1C). Therefore, we focused on the function of the operon PA4429–31 in the study.

### 3.2. The Function Analysis of PA4429–31

**Figure 2:**
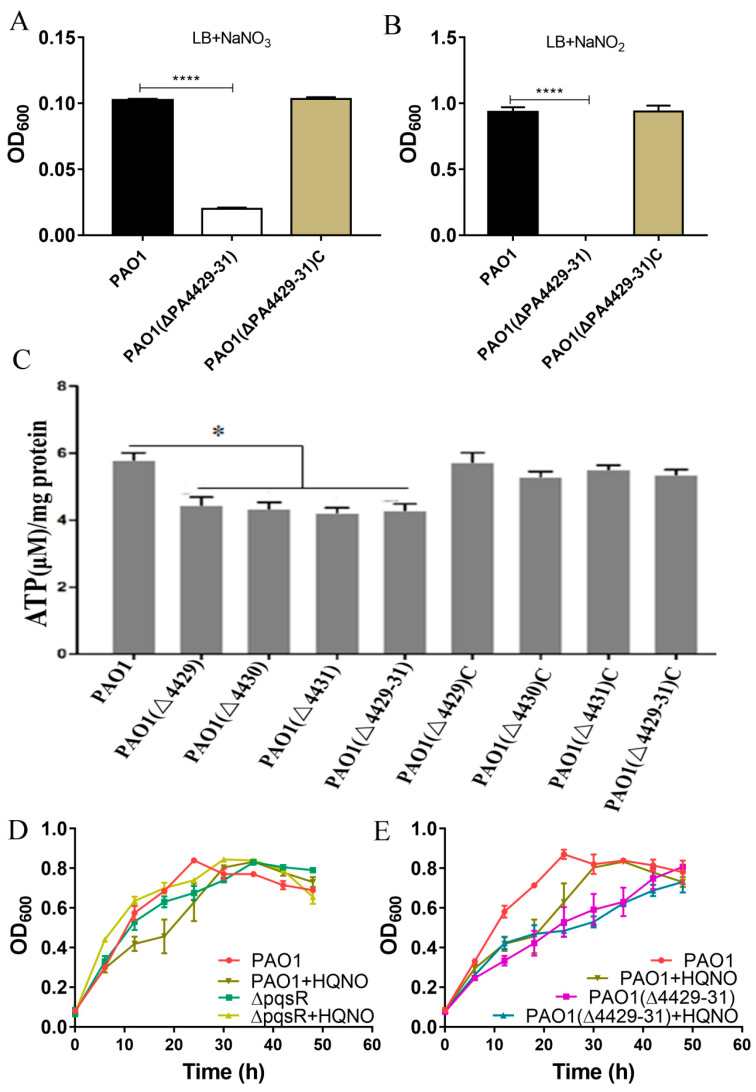
The physiological effects after PA4427–PA4431 mutation. (**A**,**B**) The effect of nitrate and nitrite on the bacterial growth. (**C**) ATP detection in the various strains. (**D**,**E**) The effect of the gene *pqsR* and the molecule HQNQ on the bacterial growth in the LB broth. The error bars represent standard errors. * *p* < 0.05. **** *p* < 0.001.

Consistent with the predicted result of the *P. aeruginosa* genome database (http://www.pseudomonas.com) (accessed on 1 May 2019), the phylogenetic tree analysis showed that that the three genes (PA4429–PA4431) were presumed to encode the cytochrome *c*1 precursor, cytochrome *b*, and iron–sulfur protein and were believed to synthesize the cytochrome *bc*1 complex (complex III) [36]. If the cytochrome *bc*1 complex is defective, PAO1 is able not to carry out aerobic respiration but can perform anaerobic respiration using nitrate and not nitrite [37]. Therefore, we measured the growth of ΔPA4429–31 under anaerobic conditions with nitrate or nitrite addition. After 18 h of incubation under anaerobic conditions, ΔPA4429–31 displayed growth when nitrate was added, although the growth was weaker compared with the wild-type PAO1 (Figure 2A). Regarding nitrite, no growth was observed for ΔPA4429–31 (Figure 2B).

During the electron transport process, cytochrome complexes I, III, and IV pump intracellular protons out of the membrane, which forms the proton driving force and catalyzes the synthesis of ATP by ATP synthase [38]. Therefore, we tested the intracellular ATP levels of ΔPA4429, ΔPA4430, ΔPA4431, and ΔPA4429–31. The results indicated that the ATP contents of the mutants were significantly lower than that of the wild type (Figure 2C).

It has been reported that HQNO (2-n-heptyl-4-hydroxyquinoline-N-oxide) is a quorum-sensing signaling molecule that specifically inhibits cytochrome *bc1* and induces programmed cell death [39]. PqsR is a transcriptional regulator of the gene cluster *pqsABCD* that is responsible for HQNO synthesis, and Δ*pqsR* cannot produce HQNO. Δ*pqsR* did not show autolysis when cultured for 48 h, but the autolysis phenomenon was recovered after exogenous HQNO addition (Figure 2D). However, we found that the PA4429–31 mutant did not exhibit autolysis within 48 h, and the autolysis was not able to recover after HQNO addition, indicating that PA4429–31 was the target cytochrome *bc1* of HQNO (Figure 2E). Taken together, all the results indicated that PA4429–31 encoded cytochrome *bc1*.

### 3.3. The Effects of PA4429–31 Deficiency on the Phenotypic Traits Related to Virulence of P. aeruginosa PAO1

**Figure 3:**
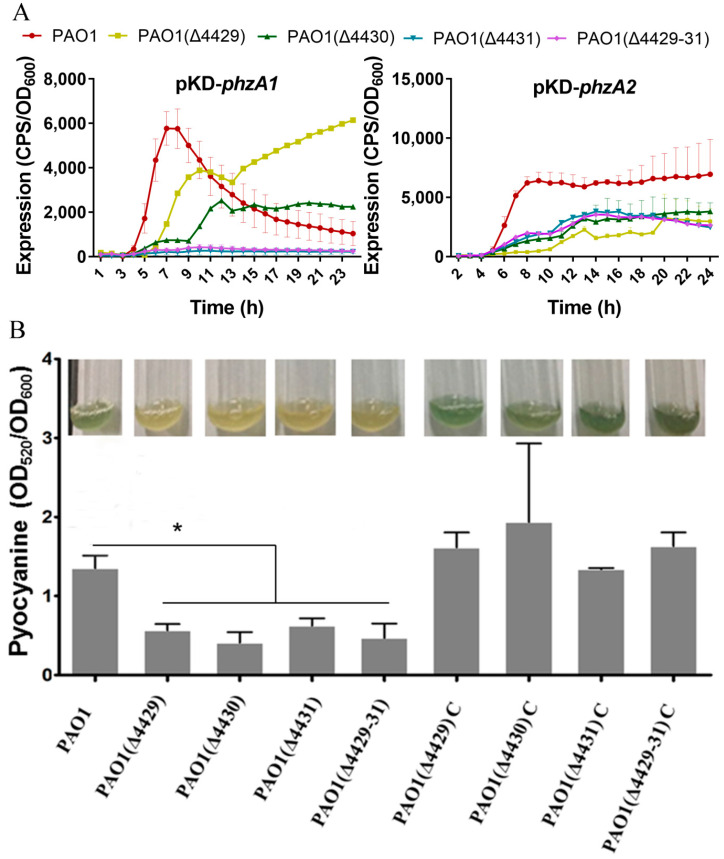
The effects of PA4429–31 deficiency on pyocyanin production. (**A**,**B**) The transcription levels of *phzA1* and *phzA2* in the wild-type strain PAO1 and the mutants. (**B**) The content of pyocyanin in the wild-type strain PAO1 and the mutants. The error bars represent standard errors. * *p* < 0.05.

Pyocyanin, a class of phenazine compounds, is a critical virulence factor secreted by *P. aeruginosa*, and *phzA1* and *phzA2* are the key genes responsible for its synthesis [40]. Therefore, the transcription of *phzA1* and *phzA2* and the pyocyanin production were examined in wild-type and ΔPA4429–31 strains. The results showed a strain-dependent difference for the *phzA1* transcription profiles. Before 11 h, the transcription levels of *phzA1* for the single- or three-mutant strains all significantly reduced, but those of the single mutants ΔPA4429 and ΔPA4430 were higher compared with the wild-type strain after 15 h (Figure 3A). For *phzA2*, the transcription levels of all the mutants of single- or three-mutant strains significantly reduced (Figure 3A). These results indicated that the gene PA4431 might be more important for pyocyanin regulation, especially for *phzA1*. The mutant produced remarkably less pyocyanin compared with the wild-type strain (Figure 3B).

**Figure 4:**
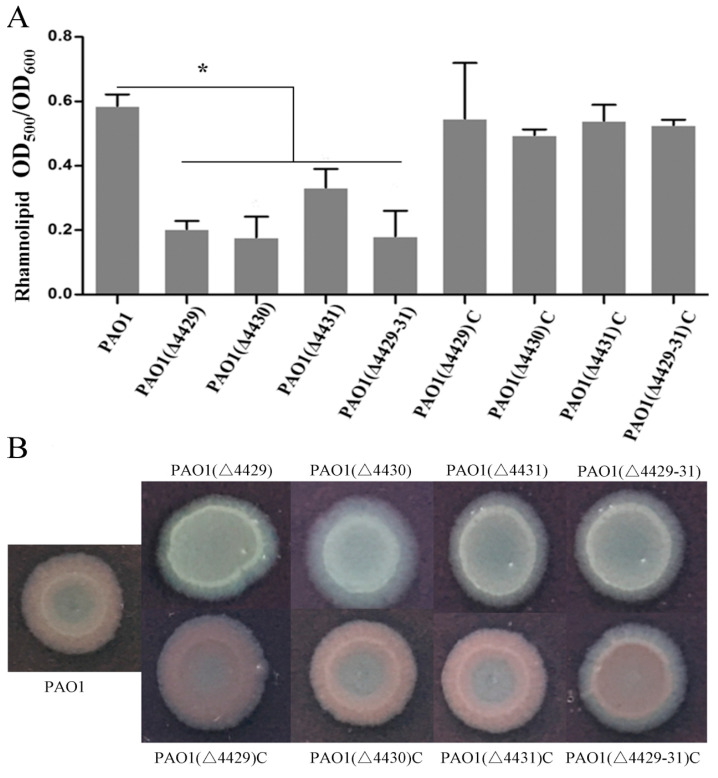
The effects of PA4429–31 deficiency on rhamnolipids (**A**) and polysaccharides (**B**) in the wild-type strain PAO1 and the mutants. (**A**) Rhamnolipid contents were normalized by the OD_600_ of the bacteria. (**B**) The strains were cultured in the plates with Congo Red. The error bars represent standard errors. * *p* < 0.05.

**Figure 5:**
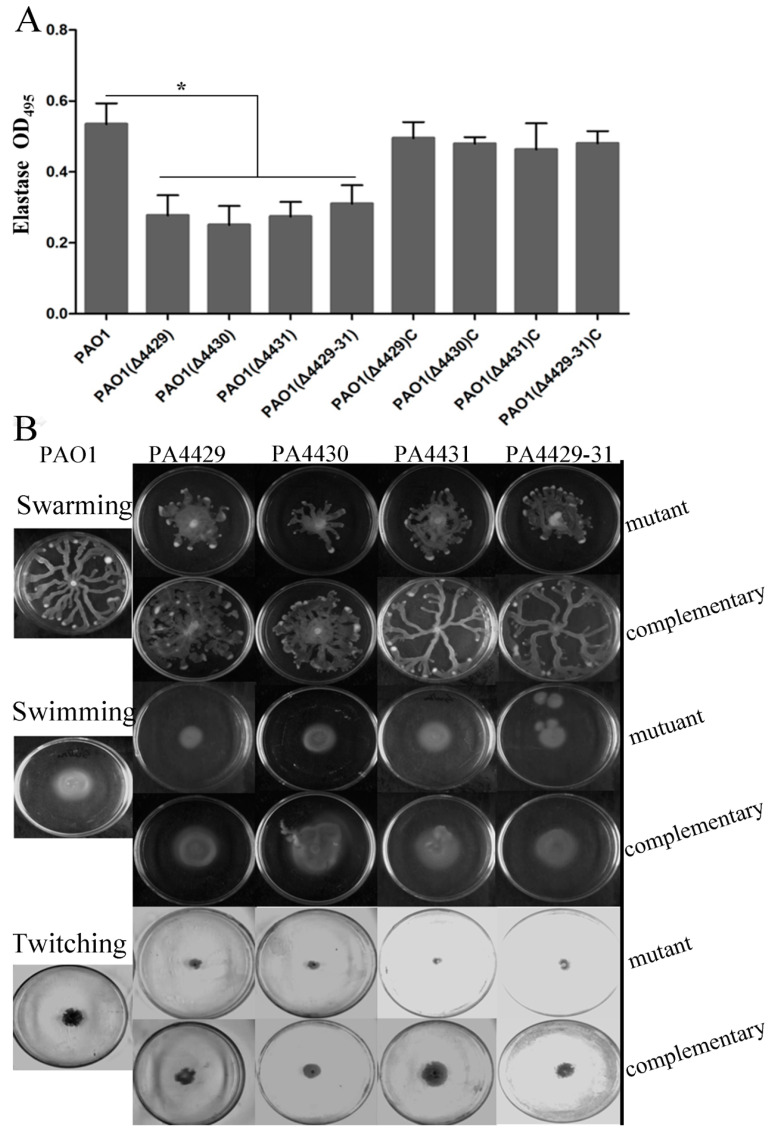
The effects of PA4429–31 deficiency on virulence and motility. (**A**) Expression levels of elastase in the wild-type strain PAO1 and the mutants. (**B**) Swarming, swimming, and twitching of the wild-type strain PAO1 and the mutants were detected. The error bars represent standard errors. * *p* < 0.05.

In addition to pyocyanin, *P. aeruginosa* also produces some other virulence factors, such as rhamnolipids, extracellular polysaccharide, and elastase [33,41,42]. These virulence factors were examined in this study. The results showed that the mutants produced much less rhamnolipid compared with the wild-type and complemented strains (Figure 4A). Extracellular polysaccharides are carbohydrate compounds, which affect bacteria attachment and biofilm formation. To explore extracellular polysaccharide synthesis, Congo Red was used in the LB agar plates. The results showed that no colonies were red in the mutants, indicating that all the mutants produced less extracellular polysaccharides compared with the wild-type strain (Figure 4B). Elastase is another important virulence factor in local infection of *P. aeruginosa*. The results showed that the elastase production of the mutants was markedly reduced compared with wild-type PAO1 (Figure 5A).

Since motility is also involved in *P. aeruginosa* infection, besides the virulence mentioned above, we further examined the effect of PA4429–31 deletion on *P. aeruginosa* motility, including swarming, swimming, and twitching. The results showed that the three kinds of motility zones in all the mutant strains were reduced by about half compared with those of the wild-type strain (Figure 5B), indicating that the motility ability for the mutant strains was impaired, but not totally inhibited.

### 3.4. The Effects of PA4429–31 on the Pathogenicity of P. aeruginosa PAO1

**Figure 6:**
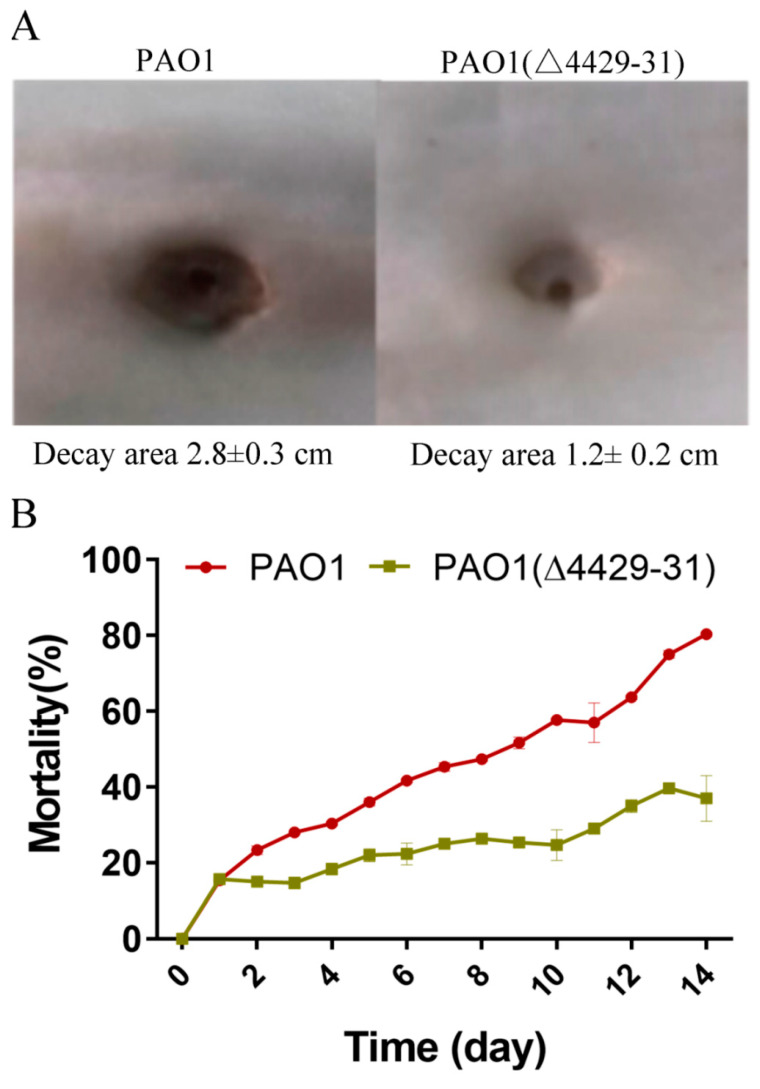
The deficiency of PA4429–31 has influenced bacterial pathogenicity. (**A**) The decay area of PAO1 and PAO1(ΔPA4429–31) in the infection model of Chinese cabbage. (**B**) The mortality of PAO1 and PAO1(ΔPA4429–31) in the infection model of *Drosophila melanogaster*.

The reduction of virulence may affect bacterial pathogenicity. Therefore, we further explored the effects of PA4429–31 deletion on the pathogenicity of *P. aeruginosa* PAO1. The cabbage and *Drosophila melanogaster* infection models were used. The results showed that in the cabbage infection model, PA4429–31deletion obviously reduced the decay area compared with that of the wild-type strain (Figure 6A). In the *Drosophila melanogaster* infection model, the mortality rate with the infection of the PA4429–31 mutant was significantly lower compared with the wild-type strain (Figure 6B). All the results indicated that the complete pathogenicity of *P. aeruginosa* PAO1 required a functional cyt*bc1*.

### 3.5. PA4429–31 Deletion Affected the Quorum Sensing and Iron Adsorption Systems of P. aeruginosa PAO1

**Figure 7:**
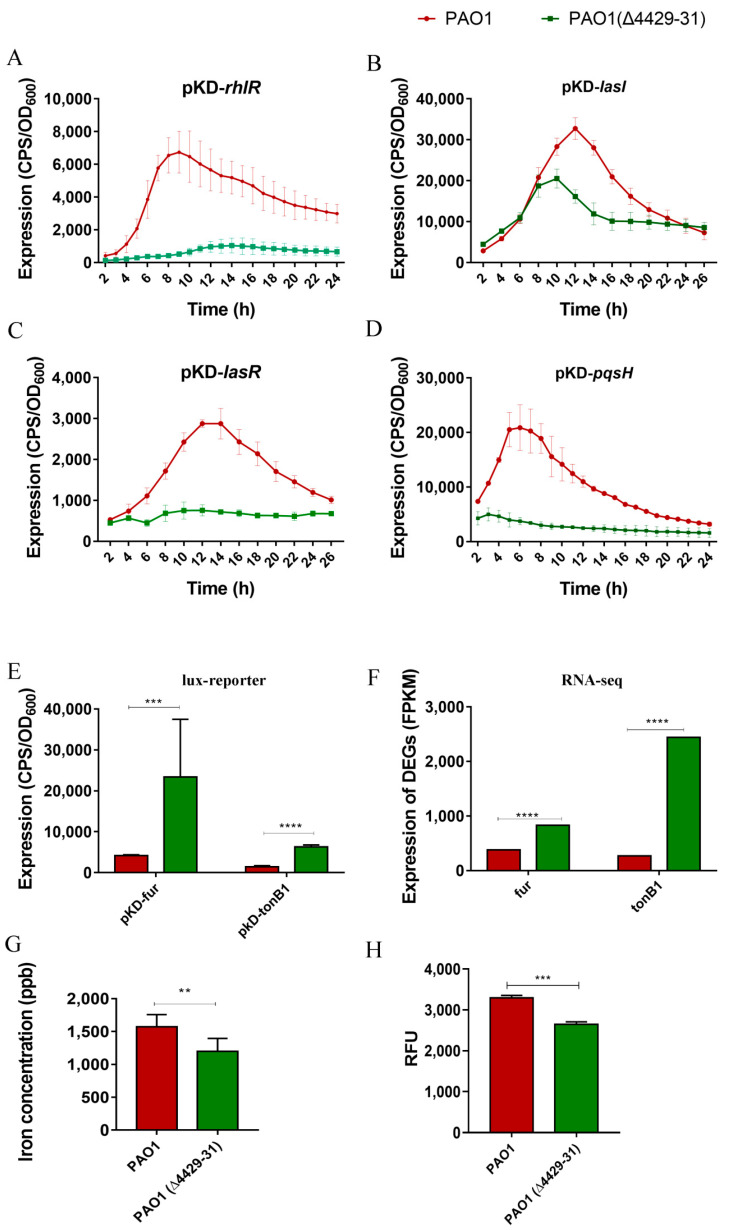
The transcriptional profiles of the genes related to the quorum sensing system, iron metabolism and the iron concentration in bacterial cells. (**A**–**D**) The transcriptional levels of *lasI, lasR, pqsH,* and *rhlR* were detected through the CPS (count per second) values. (**E**,**F**) The transcriptional levels of *tonB1* and *fur* were detected through the CPS values and RNA-Seq analysis. The FPKM (fragments per kilobase of exon model per million mapped fragments) is the normalized number of transcripts measured for each gene. (**G**) The content of iron was tested through ICP-MS in PAO1 and PAO1(Δ4429–31). (**H**) The ROS was detected by using 2’, 7’-dichlorofluorescein diacetate (carboxy-H2DCFDA) in PAO1 and PAO1(Δ4429–31). RFU: relative fluorescence units. The error bars represent standard errors. ** *p* < 0.01. *** *p* < 0.001. **** *p* < 0.0001.

To investigate the underlying mechanism of the virulence and pathogenicity reduction in the cyt*bc1* mutant, RNA-Seq analysis was performed. The results showed that the genes involved in the quorum sensing and iron adsorption systems remarkably changed (Table 4 and Table 5). These two systems have been reported to be related to the virulence and pathogenicity of *P. aeruginosa* [43,44,45], and therefore, we examined them in the next experiments. The expression of these related genes was measured using the *lux*-based reporter system. The results indicated that all the quorum sensing genes tested, including *lasR*, *lasI*, *rhlR*, and *pqsH*, were all downregulated in the PA4429–31 mutant (Figure 7A–D). The expression levels of the two key genes related to iron adsorption, *fur* and *tonB1*, remarkably increased (Figure 7E,F). In addition, the iron content of the PA4429–31mutant was obviously lower than that of the wild-type strain (Figure 6G). Taken together, it indicated that the quorum sensing and iron adsorption systems might be involved in the reduction of the virulence and pathogenicity of the cyt*bc1* mutant.

## 4. Discussion

*P. aeruginosa* has a multibranched, complex respiratory chain, which allows it to adapt to a variety of living conditions [46]. The respiratory chain is not only important for the survival of bacteria, but also has an impact on the drug resistance [47] and pathogenicity [48] of bacteria. In this study, the mutation of the respiratory chain complex III, cyt*bc*1, displayed increased resistance to aminoglycoside antibiotics and decreased contents of various virulence factors such as pyocyanin, rhamnolipid, and extracellular polysaccharides. The entry of aminoglycoside antibiotics into bacteria requires a potential difference between the inside and outside of the cell membrane [49]. The bacterial respiratory chain complex I or Fe-S cluster synthesis gene ISC mutation can increase the aminoglycoside resistance. In this study, the deletion of cyt*bc*1 caused an increase in aminoglycoside resistance, which might be due to disturbing the complex III transmembrane transport proton or reducing the synthesis of complex I and complex II, resulting in the loss of iron–sulfur protein. In addition, many studies have found that antibiotics can induce the production of ROS and lead to bacterial death. In this study, it was found that the ROS level of the cyt*bc*1 mutant was a bit lower than that of the wild-type strain (Figure 7H), and therefore, this situation might also lead to an increase in antibiotic resistance for the mutants.

The most obvious change in the *P. aeruginosa* cyt*bc*1 mutant was the reduction of pyocyanin, rhamnolipid production and extracellular polysaccharides. *P. aeruginosa* has the ability to produce a pigmented secondary metabolite: phenazine. Pyocyanin, one kind of phenazine, is a critical virulence factor in *P. aeruginosa* during chronic infection [50]. It appeared that the reduction of pyocyanin production was mediated by the downregulation of the *phzA1* and *phzA2* operons, indicating that cyt*bc*1 affected pyocyanin production by directly regulating the *phz* genes. Cyt*bc*1 might also directly affect the synthesis of rhamnolipids and polysaccharides. However, it is well known that the quorum sensing system controls the production of virulence factors in *P. aeruginosa.* The reduced expression of quorum sensing-related genes, such as *lasI*, *lasR*, *rhlR*, and *pqsH*, in the cyt*bc*1 mutant indicated that the cyt*bc*1 indirectly affected virulence factor production through the quorum sensing systems. Exopolysaccharides, pyocyanin, and rhamnolipids all impact the formation of *P. aeruginosa* biofilm, besides virulence. Biofilm formation is an advantage in many infection situations and enhances the bacterial ability to resist antibiotics and harsh environmental conditions [50]. Therefore, cyt*bc*1 might be a target for biofilm infection treatment.

Taken together, the work illustrated that the influence of cyt*bc*1 deficiency was multifarious, which increased the antibiotic resistance but reduced the virulence and pathogenicity. It also affected quorum sensing and iron adsorption, and in return, both changed many phenotypes of this bacterium. These results help to comprehensively understand the function of the respiratory chain and develop new drug targets for *P. aeruginosa* infection.

## Figures and Tables

**Table 1:** The strains and plasmids used in this study.

Strains or Plasmids	Genotype or Phenotype	References
Strains		
***E. coli***		
DH10B	F *mcrA* Δ(*mrr-hsdRMS-mcrBC*) F 80*lacZ*Δ*M15* Δ*lacX74 recA1 ara*Δ*139*Δ(*ara-leu*)*7697 galU galK rpsL* (Str^R^) *endA1 nupG*	Invitrogen
*P. aeruginosa*		
PAO1	Wild type	This lab
PAO1(Δ4429)	PA4429 deletion mutant of PAO1	This study
PAO1(Δ4430)	PA4430 deletion mutant of PAO1	This study
PAO1(Δ4431)	PA4431 deletion mutant of PAO1	This study
PAO1(Δ4429–31)	PA4429–31 deletion mutant of PAO1	This study
PAO1(Δ4429)C1	PAO1(Δ4429) complement contains pAK4429	This study
PAO1(Δ4429)C2	PAO1(Δ4429) complement contains pAK4429–31	This study
PAO1(Δ4430)C1	PAO1(Δ4430) complement contains pAK4430	This study
PAO1(Δ4430)C2	PAO1(Δ4430) complement contains pAK4429–31	This study
PAO1(Δ4431)C1	PAO1(Δ4431) complement contains pAK4431	This study
PAO1(Δ4431)C2	PAO1(Δ4431) complement contains pAK4429–31	This study
PAO1(Δ4429–31)C	PAO1(Δ4429–31) complement contains pA4429–31	This study
Plasmids		
pEX18Tc	*oriT*^+^*sacB*^+^ gene replacement vector with mμltiple cloning site from pUC18; Tc^r^	This lab
pRK2013	Broad-host-range helper vector; Tra^+^, Kan^R^	This lab
pAK1900	Multicopy *E. coli-P. aeruginosa* shuttle vectorwith an MCS within *lacZ* fragment	This lab
pMS402	Expression reporter plasmid carrying the promoter less *luxCDABE,* Kan^R^,Tmp^R^	This lab
pKD-*phzA1*	pMS402 contains *phzA1* promoter region	This study
pKD-*phzA2*	pMS402 contains *phzA2* promoter region	This study
pKD-*lasI*	pMS402 contains *lasI* promoter region	This study
pKD-*lasR*	pMS402 contains *lasR* promoter region	This study
pKD-*rhlR*	pMS402 contains *rhlR* promoter region	This study
pKD-*rhlI*	pMS402 contains *rhlI* promoter region	This study
pKD-*pqsH*	pMS402 contains *pqsH* promoter region	This study
pKD-*fur*	pMS402 contains *fur* promoter region	This study
pKD-*tonB1*	pMS402 contains *tonB1*promoter region	This study

**Table 2:** The primers used in this study.

Primer	Sequence (5′→3′)	Restriction Site
PA4429-UP-S	CGTGAATTCTCAACGGAAGACAGGCT	*Eco*R I
PA4429-UP-A	CGTGAGCTCTCTTCGTATTCGCCTATC	*Sac* I
PA4429-D-S	AGCGAGCTCACATGATCCAGTTGCGG	*Sac* I
PA4429-D-A	AATGGTACCTCGGCGTGGACCAGGAGA	*Kpn* I
PA4430-UP-S	AGTGAATTCGGGTCGGAATAGCAGGTC	*Eco*R I
PA4430-UP-A	CTTGAGCTCCTGATGCCGTTCTACACC	*Sac* I
PA4430-D-S	AGTGAGCTCTTGACCAGCACCAGCAGC	*Sac* I
PA4430-D-A	TATCCCGGGTAAGAAAGTCGGTCTGCG	*Sma* I
PA4431-UP-S	GTCGAATTCTCATCAGCCAGTCACCCT	*Eco*R I
PA4431-UP-A	ATAGAGCTCCGTGGACCAGGAGAAAGC	*Sac* I
PA4431-D-S	CGAGAGCTCCATTCACGCCGTCATTA	*Sac* I
PA4431-D-A PA4429–31-UP-S PA4429–31-UP-A PA4429–31-D-S PA4429–31-D-A	GCAGGTACCAAGACCACCGACAAGATG CCTGAGCTCCGATATGTATCGCAAGCTG AAGTCTAGAGCCTGCATTCACGCCGTC CTGTCTAGATAACCCGCACGTTGGTC GAACTGCAGACATTGAGCACGATCTG	*Kpn* I *Sac* I *Xba* I *Xba* I *Pst* I
For Complemented Strains		
PA4429-F	ATCGAAGCTTAGGCTGCCAGGTTAA	*Hin*d Ⅲ
PA4429-R	AATAGGATCCGATCCGAATATCGA	*Bam*H I
PA4430-F	CGCAAGCTTATACGTTCGATGCTGAC	*Hin*d Ⅲ
PA4430-R	CCTTCTAGAGCCAGAATCAGTGCAGC	*Xba* I
PA4431-F	TGGGCATGCGTCAGGCTATTACCTTG	*Sph* I
PA4431-R	GGCGTCGACTCTTCCCACATCTTGG	*Spl* I
PA4429–31-F	AGTTCTAGACGGCGTGAATGCAGGC	*Xba* I
PA4429–31-R	AAGGAGCTCACCAACGTGCGGGTTAG	*Sac* I
RT-PCR		
random PCR primer F	GTGCTGACCCCGGATGAAGTGGTTCGCATC	None
random PCR primer R	GGATGCGTCTAAAAGCCTGC	None
r1	GACATTGAGCACGATCTG	None
r2	ACAAGCTGACCTGCTATTC	None
r3	ACATGATCCAGTTGCGG	None
r4	CGTGGACCAGGAGAAAGC	None
r5	GGGTCGGAATAGCAGGTC	None
r6	GAACCTGGTGACCTTCCTG	None

**Table 3:** MIC values of the mutants against four aminoglycoside antibiotics.

The Strain	MIC (μg/mL)			
	Kan	Gm	Tob	Amk
PAO1	64	32	2	1
PAO1(△4429)	256	128	8	4
PAO1(△4430)	256	128	8	4
PAO1(△4431)	256	128	8	4

**Table 4:** Differential expression genes involved in the quorum sensing system.

Gene ID	Gene Name	Product	Fold Change
PA5531	*tonB1*	Transporter TonB	3.18
PA4764	*fur*	Ferric uptake regulation protein	1.12
PA2426	*pvds*	Extracytoplasmic-function sigma-70 factor	3.27
PA2398	*fpvA*	Ferripyoverdine receptor	2.86
PA2386	*pvdA*	L-ornithine, N5-oxygenase, pyoverdine produce	2.25
PA1911	*fecR*	Iron dicitrate transport regulator FecR	4.86
PA3209	*ykgJ*	Zinc/iron-chelating, domain-containing protein	−12.95
PA3518		Iron-containing redox enzyme family protein	−11.80
PA4159	*fepB*	Transporter periplasmic-binding protein	2.14
PA4191		Iron oxidase	−9.19
PA1910	*fcuA*	Ferric-mycobactin receptor FemA	2.01
PA2466	*foxA*	Ferrioxamine receptor FoxA	2.46
PA3812	*iscA*	Iron-binding protein IscA	2.31
PA3814	*iscS*	Cysteine desulfurase	2.35
PA5483	*algB*	Two-component response regulator AlgB	−2.06
PA2384		Ferric uptake regulator, Fur family	6.59
PA3901	*fecA*	Fe(III) dicitrate transporter FecA	3.41

**Table 5:** Differential expression genes involved in the iron metabolism.

Gene ID	Gene Name	Product	Fold Change
PA0609	*trpE*	Anthranilate synthase component I	−1.15
A0649	*trpG*	Anthranilate synthase component II	−1.17
PA0996	*pqsA*	Anthranilate--CoA ligase	−1.01
PA0997	*pqsB*	PqsB	−1.48
PA0998	*pqsC*	Beta-keto-ACP synthase	−1.17
PA1303	*lepB*	Signal peptidase	−13.23
PA1432	*lasI*	Acyl-homoserine-lactone synthase	−1.05
PA1871	*lasA*	Protease LasA	−2.28
PA1901	*phzC*	Phenazine biosynthesis protein PhzC	−1.14
PA2570	*lecA*	PA-I galactophilic lectin	1.24
PA3478		Rhamnosyltransferase subunit B	−1.38
PA3724	*lasB*	Elastase LasB	−1.08
PA4206	*mdtA*	Resistance-nodulation-cell division (RND) efflux membrane Fusion protein	−1.66
PA4210	*phzA1*	Phenazine biosynthesis protein	−2.62
PA4211	*phzB1*	Phenazine biosynthesis protein phzB 1	−1.95
PA4212	*phzC*	Phenazine biosynthesis protein PhzC	−1.16
PA4815		Integral membrane protein	1.78
PA4944	*hfq*	RNA-binding protein Hfq	1.06
